# Association between dietary intake and symptoms of depression and anxiety in pregnant women: Evidence from a community‐based observational study

**DOI:** 10.1002/fsn3.3675

**Published:** 2023-09-21

**Authors:** Yunhan Yu, Wensu Zhou, Xidi Zhu, Zhao Hu, Shaojie Li, Baohua Zheng, Huilan Xu, Wei Long, Xiyue Xiong

**Affiliations:** ^1^ Medical Administration Division Hengyang Central Hospital Hengyang China; ^2^ Department of Medical Statistics, School of Public Health Sun Yat‐sen University Guangzhou China; ^3^ Department of Social Medicine and Health Management, Xiangya School of Public Health Central South University Changsha China; ^4^ Department of Epidemiology and Health Statistics, Xiangya School of Public Health Central South University Changsha China; ^5^ NHC Key Laboratory of Birth Defect for Research and Prevention Hunan Provincial Maternal and Child Health Care Hospital Changsha China

**Keywords:** dietary intake, mental health symptoms, pregnant women, prevention

## Abstract

Dietary intake is considered as a crucial factor affecting mental health symptoms, particularly depression and anxiety symptoms, especially in the case of pregnant women. This study explored the role of dietary intake in depression and anxiety symptoms of pregnant women and provided evidence for primary care interventions. We enrolled 806 pregnant women in their third trimester from 14 communities in Hengyang City, Hunan Province, China, from July 2019 to September 2019. The Chinese version of the Patient Health Questionnaire‐9 (PHQ‐9) and the Generalized Anxiety Disorder questionnaire‐7 (GAD‐7) were used to assess depression and anxiety symptoms. Dietary intake, demographic characteristics, BMI, and pregnancy characteristics were collected using a self‐designed, structural questionnaire. A covariate‐adjusted logistic regression was conducted to examine the relationship between mental health symptoms and dietary intake. The prevalence of anxiety and depression symptoms in our population were 7.7% (95% CI: 5.9%–9.5%) and 9.2% (95% CI: 7.2%–11.2%), respectively. Women consuming eggs and egg products once a week (OR: 3.688, 95% CI: 1.476–9.215) were more likely to have depression symptom than consumed eggs and egg products once or more per day. Consuming green leafy vegetable <2–3 times per month had a significantly greater risk for depression symptoms than consuming the same once or more per day (OR: 3.450, 95% CI: 1.145–10.393). Women who consumed eggs and egg products 2–3 times a week had an increased likelihood of experiencing anxiety symptoms (OR: 2.253, 95% CI: 1.049–4.837). Anxiety symptoms in women consuming green leafy vegetables <2–3 times per month probably increased by 3.988 times (95% CI: 1.327–11.985) compared with women consuming the same once or more per day. Consuming salted and smoked food <2–3 times per month was protective against anxiety symptoms (OR: 0.181, 95% CI: 0.040–0.828) than consuming the same every day. Implementing interventions to promote healthy dietary among pregnant women is crucial due to its association with mental health. However, future researches are warranted to confirm the reliability and causal association obtained in this study.

## Graphical abstract

The study was a community‐based investigation that enrolled more than 800 pregnant women. The study result found the dietary pattern among pregnancy was associated with mental health symptoms.

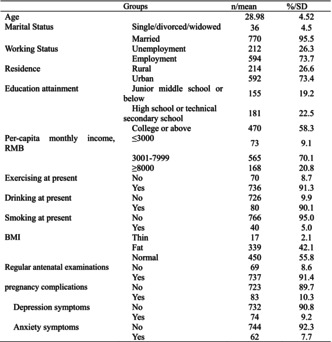

## 1 INTRODUCTION

Mental health in pregnant women is a global public health problem, and it plays a vital role in their quality of life and the fetal growth and development (de Jesus Silva & Alves Nogueira, 2016; Tang et al., 2019; Uguz et al., 2019). Depression and anxiety symptoms are commonly reported during pregnancy according to scholarly researches (Cameron et al., 2016). The prevalence of prenatal depression and anxiety symptoms in each trimester of pregnancy in low‐ and middle‐income countries (LMICs) was estimated to be 5.19%–46.8% (Dennis et al., 2017; Ma et al., 2019) and 11.1%–34.4% (Phoosuwan et al., 2018; Tang et al., 2019), respectively. A systematic review comparing LMICs to high‐income countries (HICs) highlighted a higher prevalence of depression and anxiety symptoms among pregnant women in LMICs (Fekadu Dadi et al., 2020; Fisher et al., 2012). Additionally, researchers have emphasized that mental health symptom prevalence tends to be higher during the third trimester compared to other phases of pregnancy (Ayano et al., 2019; Dennis et al., 2017; van de Loo et al., 2018). These symptoms can have adverse health outcomes for both the mother and the fetus (Uguz et al., 2019). It is increasingly important to identify factors related to the reduction of such symptoms in pregnant women.

During pregnancy, dietary intake is an important and fundamental source of nutrition for the mother and the fetus. Numerous studies conducted in the past decades have explored the relationship between dietary intake and mental health symptoms in pregnant women (Khan et al., 2020; Silva et al., 2019; Therrien et al., 2021). For instance, a cross‐sectional survey involving 712 pregnant women conducted in Brazil found that low level of fruit intake and high intake of sweets were associated with symptoms of depression, and low intake of beans was related to increased anxiety symptoms (Paskulin et al., 2017). In the study by Baskin et al., unhealthy dietary intake was probably associated with depression symptoms in the third trimester (Baskin et al., 2017). Similar conclusions could also be confirmed in studies from Brazil, Japan, the US, and Greece (Carter et al., 2000; Chatzi et al., 2011; Miyake et al., 2013; Vilela et al., 2015). However, in pregnant women, the association between dietary intake and emotional symptoms has not always showed up consistently in previous studies (Chong et al., 2014; Lukose et al., 2014). For example, in the study by Lukose et al., common nutrient intake was uncorrelated with depression symptoms in Indian pregnant women (Lukose et al., 2014). In terms of biology, micronutrients derived from dietary elements, such as chromium, zinc, iron, copper, calcium, magnesium, and tryptophan could mediate the physiological function and neurotransmitter metabolism of the brain, affecting the emotions of individuals (Silva et al., 2019; Sparling et al., 2016). Additionally, an insufficient and unhealthy diet could contribute to increased pressure for pregnant women (Boutte et al., 2021). Thus, the influence of dietary on mental health among pregnant women are pronounced.

However, to date, there are several gaps that should be further discussed. Though prior studies have reported a link between dietary intake and depression symptoms in pregnant women, there is limited evidence regarding the impact of dietary intake on prenatal anxiety symptoms (Silva et al., 2019). Few studies focused on the third trimester, even though this period had a relatively higher prevalence of mental problems than other periods. Moreover, evidence about whether mental health symptoms could be attributed to dietary intake in the Chinese population is still lacking. These associations reported in Western countries may not be applicable to the Chinese population due to differences in lifestyle, culture, and economic development. Most importantly, dietary patterns are identified as modifiable lifestyle factors for pregnant women and require more timely intervention during the entire pregnancy period.

Hence, this study aims to examine the correlation between dietary intake and emotional symptoms among pregnant women through a community‐based investigation. We aspire that our evidence may assist pregnant women in combating emotional symptoms.

## 2 METHOD

### 2.1 Study design and population

**FIGURE 1:**
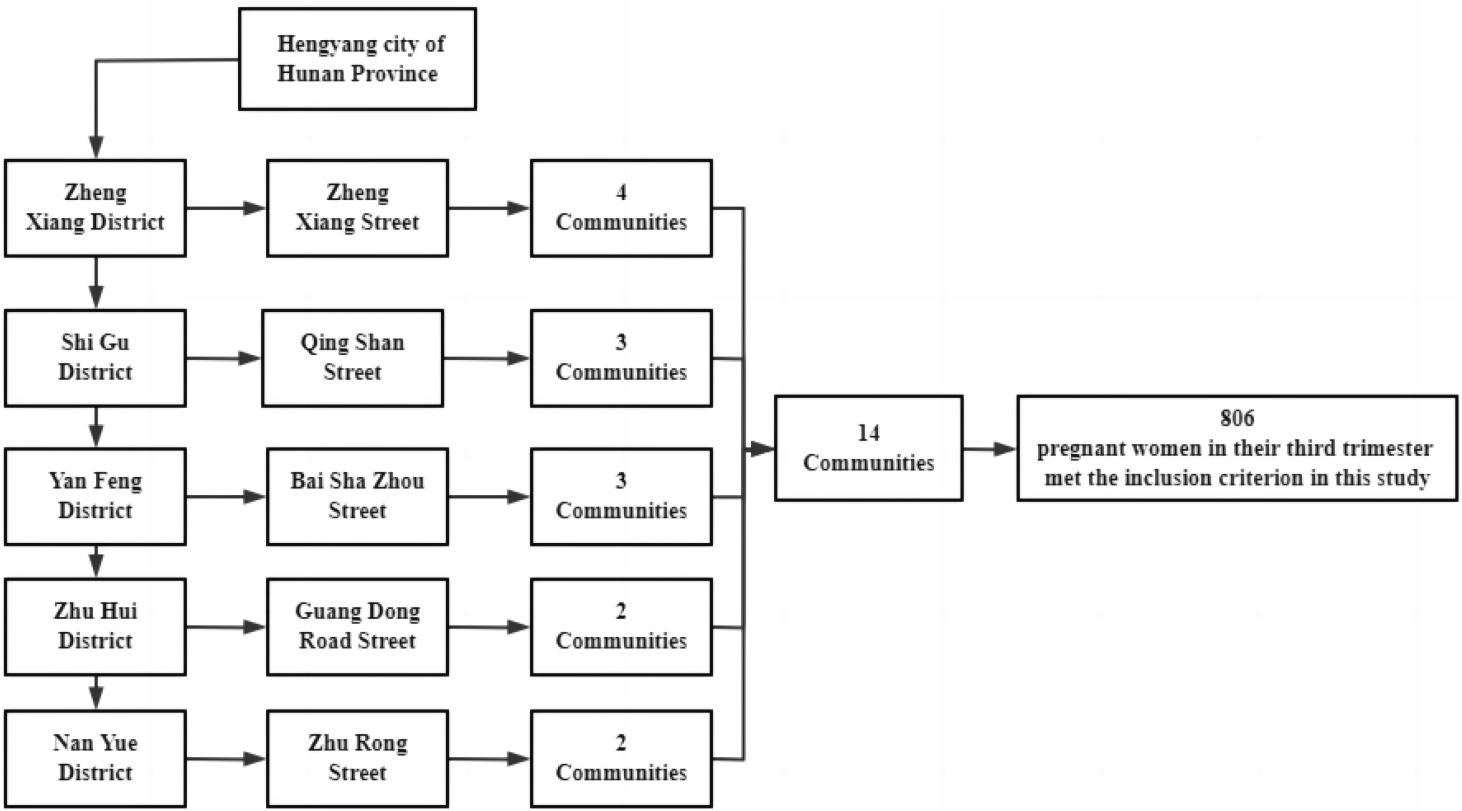
Sampling strategy for the 10 communities of Hengyang city in our study.

This was a cross‐sectional study based on a community conducted in Hengyang City, Hunan Province, China. First, the researchers randomly selected one street from the five districts of the city. Fourteen communities were randomly selected at a ratio of 1:3 (district: street), including four communities from Zhengxiang Street, three from Qingshan Street, three from Baishazhou Street, two from Guangdong Road Street, and two from Zhurong Street. A total of 813 pregnant women in their third trimester were recruited from these communities between July 2019 and September 2019. The inclusion criteria for the participants in this study were as follows: (1) age over 18 years, (2) Voluntary participation in surveys, (3) pregnant women in the community health center, (4) pregnant women without previous medical history, and (5) completed interviews with complete information. After excluding seven women who did not meet the inclusion criteria, 806 participants were included in the final statistical analysis. The flow chart was presented in Figure 1. Information about the demographic characteristics, dietary intake, behavioral lifestyle, BMI, and pregnancy complications were extracted from a self‐designed, structural questionnaire. All participants were interviewed in‐person for about 20 min by trained graduate students from the Xiangya School of Public Health, Central South University. All participants provided signed, informed consent.

### 2.2 Assessment of dietary intake frequency

Referring to previous studies, this study used food frequency questionnaire to assess dietary intakes, this questionnaire was adapted from Chinese Longitudinal Healthy Longevity Survey (CLHLS) (Shi et al., 2015). Participants were asked to recall their dietary intake of the previous month and completed this food frequency questionnaire about specific food. Scholars have pointed out that assessing the level estimates and ranking individuals on intakes, rather than precise levels of intake, has good reliability and can simplify individual dietary intake assessment (Yaroch et al., 2012). The participants filled in information about dietary intake of cereals (rice and maize), fruits (fresh fruit and fruit juice), green leafy vegetables, milk and its products, eggs and its products, aquatic products (fish, shrimp, crab, shellfish), bean and its products, meat, and salted and smoked food in the previous month. Dietary intake was categorized into four groups: ≥1 time/day (reference), 2–3 times/week, 1 time/week, and <2–3 times/month except cereals (rice and maize). Cereal is a staple food in the diet of Chinese people. Hence, cereal intake was divided into three groups as follows: 1 time/day (reference), 1–3 times /week, and 2–3 times/month.

### 2.3 Assessment of emotional symptoms

The Chinese version of the Patient Health Questionnaire‐9 (PHQ‐9) and Generalized Anxiety Disorder questionnaire‐7 (GAD‐7) were used to assess depression and anxiety symptoms in pregnant women, respectively. The PHQ‐9 is a 9‐item screening scale for major depressive disorder experienced in the previous 2 weeks. According to the frequency of symptoms, each item is rated from “0” (not at all), “1” (several days), “2” (more than half the days) to “3” (almost every day). Total scores ranged from 0 to 27. The GAD‐7 scale is a simple tool with seven items for screening anxiety disorders experienced in the previous 2 weeks, which is designed based on the DSM‐IV diagnostic criteria. Each item is rated on a scale from “0” (none), “1” (a few days), “2” (more than half the time), to “3” (almost every day). The scores ranged from 0 to 21. Individuals with higher scores can be considered to have more severe emotional symptoms. The screening cut‐off score was 10 points for depression and anxiety symptoms (Kroenke et al., 2001; Spitzer et al., 2006). Previous studies confirmed that the GAD‐7 and PHQ‐9 were suitable for screening emotional symptoms in the Chinese population because they have good reliability and validity (Wang et al., 2014; Zhang et al., 2021). In our study, the Cronbach's α coefficients for the GAD‐7 and PHQ‐9 were 0.882 and 0.773, respectively. The depression and anxiety symptoms were binary variables in this study coded as “0” (no symptoms) and “1” (has symptoms).

### 2.4 Covariates

The following covariates were acquired from a self‐designed questionnaire: age (continuous variable), education level (college or above and high school or below), residence (rural or urban), marital status (married and divorced/widowed/single), employment (yes or no), and per‐capita monthly income (≥8000, 3001–7999 and ≤3000 RMB). Current lifestyle included smoking habits (yes, former, and no), drinking (yes or no), and exercise habits (yes or no). The BMI was categorized as thin (18.5 kg/m^2^), normal (18.5–23.9 kg/m^2^), and fat (≥24 kg/m^2^; Yu et al., 2020), regular antenatal examinations (yes or no), pregnancy complications (yes or no) were included as covariates.

### 2.5 Statistical analysis

Mean and standard deviation (SD) were reported for continuous variables (age). Frequency and percentage were expressed as categorical variables. The impact of dietary intake on mental health symptoms was compared using a binary logistic regression model with a logit distribution to derive odds risks (ORs) and 95% CIs. Model 1 included dietary frequency of cereals (rice and maize), fruits (fresh fruit and fruit juice), green leafy vegetables, milk and milk products, eggs and egg products, aquatic products (fish, shrimp, crab, shellfish), bean and bean products, meat, and salted and smoked food. Model 2 was adjusted for covariates (age, educational attainment, residence, marital status, employment, per‐capita monthly income, current smoking habits, drinking, exercise habits, BMI, and pregnancy complications). Analysis were performed using IBM SPSS Version 25 (IBM) and statistical significance defined as two‐sided with a *p*‐value <.05.

## 3 RESULTS

### 3.1 Characteristics of study participants

Table 1 presents the characteristics of the 806 participants. The average age of pregnant women was 28.98 (SD = 4.52) years. The majority of women were under urban registration (73.4%), were married (95.5%), employed (73.7%), and had attained college or higher educational degrees (58.3%); 70.1% had a per‐capita monthly income between 3000 and 7000 RMB. Most of them lived a healthy lifestyle, with only 5% who smoked, 9.9% drank, and 8.7% did not exercise. 55.8% of them had normal BMI and 42.1% were fat, 91.4% received regular antenatal examinations, and 10.3% had pregnancy complications. The prevalence of anxiety and depression symptoms was 7.7% (95% CI: 5.9%–9.5%) and 9.2% (95% CI: 7.2%–11.2%), respectively.

**TABLE 1:** The characteristics of general information in pregnant women.

Groups	*n*/mean	%/SD
Age	28.98	4.52
*Marital status*		
Single/divorced/widowed	36	4.5
Married	770	95.5
*Working status*		
Unemployment	212	26.3
Employment	594	73.7
*Residence*		
Rural	214	26.6
Urban	592	73.4
*Education attainment*		
Junior middle school or below	155	19.2
High school or technical secondary school	181	22.5
College or above	470	58.3
*Per‐capita monthly income*, *RMB*		
≤3000	73	9.1
3001–7999	565	70.1
≥8000	168	20.8
*Exercising at present*		
No	70	8.7
Yes	736	91.3
*Drinking at present*		
No	726	9.9
Yes	80	90.1
*Smoking at present*		
No	766	95.0
Yes	40	5.0
*BMI*		
Thin	17	2.1
Fat	339	42.1
Normal	450	55.8
*Regular antenatal examinations*		
No	69	8.6
Yes	737	91.4
*Pregnancy complications*		
No	723	89.7
Yes	83	10.3
*Depression symptoms*		
No	732	90.8
Yes	74	9.2
*Anxiety symptoms*		
No	744	92.3
Yes	62	7.7

### 3.2 Characteristics of dietary intake

Table 2 shows an overview of the characteristics of the dietary intake of the women. Over half of the percentage of women consumed cereals (65.1%), eggs and egg products (53.7%), green leafy vegetables (63.3%), and fruits (69.7%) once or more per day. Further, 41.3% consumed milk and its products once or more per day; 15.5%, 17.6%, and 25.2% consumed aquatic products, beans and their products, and meat once or more per day. However, there was a lower percentage (2.0%) of participants who consumed salted and smoked foods more than once a week.

**TABLE 2:** The characteristics of dietary intake in pregnant women.

Variables	Groups	*n*	%
Cereals (rice and maize)	≥1 time/day	525	65.2
	2–3 times /week	105	13.0
	1 times /week	176	21.8
Milk and milk products	≥1 time/day	333	41.3
	2–3 times /week	264	32.8
	1 times /week	83	10.3
	<2–3 times/month	126	15.6
Eggs and egg products	≥1 time/day	433	53.7
	2–3 times /week	262	32.5
	1 times /week	52	6.5
	<2–3 times/month	59	7.3
Fruits (fresh fruit and fruit juice)	≥1 time/day	562	69.7
	2–3 times /week	167	20.7
	1 times /week	36	4.5
	<2–3 times/month	41	5.1
Green leafy vegetables	≥1 time/day	510	63.3
	2–3 times /week	176	21.8
	1 times /week	59	7.3
	<2–3 times/month	61	7.6
Aquatic products (fish, shrimp, crab, shellfish)	≥1 time/day	125	15.5
	2–3 times /week	302	37.5
	1 times /week	179	22.2
	<2–3 times/month	200	24.8
Bean and its products	≥1 time/day	142	17.6
	2–3 times /week	328	40.7
	1 times /week	144	17.9
	<2–3 times/month	192	23.8
Meat	≥1 time/day	203	25.2
	2–3 times /week	260	32.2
	1 times /week	120	14.9
	<2–3 times/month	223	27.7
Salted and smoked food	≥1 time/day	16	2.0
	2–3 times /week	81	10.0
	1 times /week	77	9.6
	<2–3 times/month	632	78.4

### 3.3 Dietary intake and depression symptoms

Table 3 presents the association between dietary intake and depression symptoms in pregnant women. After adjusting for covariates, compared to the participants consuming eggs and egg products once or more per day, the risk of depression symptoms was 3.688 (95% CI: 1.476–9.215) for women consuming the same only once a week. Women consuming green leafy vegetable <2–3 times per month were more likely to have depressive symptoms than those who consumed green leafy vegetables once or more per day (OR: 3.450, 95% CI: 1.145–10.393). The fully adjusted binary logistic regression model had a goodness‐of‐fit using the Hosmer–Lemeshow test (χ^2^ = 6.941, *p* = .543).

**TABLE 3:** The association between depression symptoms and dietary intake.

Variables	Groups	Model 1		Model 2	
		OR	95% CI	OR	95% CI
Cereals (rice and maize)	≥1 time/day	1		1	
	2–3 times /week	1.269	0.630–2.554	1.316	0.644–2.692
	1 times /week	0.562	0.275–1.151	0.556	0.268–1.156
Milk and milk products	≥1 time/day	1		1	
	2–3 times /week	0.824	0.410–1.655	0.802	0.392–1.638
	1 times /week	0.979	0.388–2.472	0.995	0.383–2.588
	<2–3 times/month	1.896	0.873–4.120	1.945	0.862–4.389
Eggs and egg products	≥1 time/day	1		1	
	2–3 times /week	1.332	0.679–2.612	1.285	0.641–2.579
	1 times /week	**3.710**	**1.523–9.037**	**3.688**	**1.476–9.215**
	<2–3 times/month	0.627	0.171–2.297	0.578	0.154–2.171
Fruits (fresh fruit and fruit juice)	≥1 time/day	1		1	
	2–3 times /week	1.224	0.561–2.672	1.100	0.495–2.443
	1 times /week	1.428	0.457–4.464	1.391	0.427–4.533
	<2–3 times/month	0.511	0.120–2.173	0.489	0.110–2.170
Green leafy vegetables	≥1 time/day	1		1	
	2–3 times /week	1.209	0.561–2.605	1.185	0.536–2.617
	1 times /week	1.434	0.487–4.220	1.119	0.363–3.448
	<2–3 times/month	**3.800**	**1.323–10.915**	**3.450**	**1.145–10.393**
Aquatic products (fish, shrimp, crab, shellfish)	≥1 time/day	1		1	
	2–3 times /week	0.650	0.251–1.681	0.732	0.274–1.951
	1 times /week	0.892	0.320–2.487	0.945	0.330–2.710
	<2–3 times/month	0.558	0.189–1.651	0.581	0.190–1.783
Bean and bean products	≥1 time/day	1		1	
	2–3 times /week	1.611	0.619–4.191	1.671	0.628–4.445
	1 times /week	1.659	0.571–4.818	1.748	0.582–5.250
	<2–3 times/month	0.825	0.264–2.575	0.849	0.264–2.730
Meat	≥1 time/day	1		1	
	2–3 times /week	1.497	0.722–3.102	1.583	0.746–3.361
	1 times /week	1.112	0.463–2.667	1.129	0.461–2.769
	<2–3 times/month	0.834	0.344–2.023	0.809	0.327–2.001
Salted and smoked food	≥1 time/day	1		1	
	2–3 times /week	0.510	0.118–2.205	0.422	0.092–1.933
	1 times /week	0.279	0.059–1.321	0.240	0.048–1.197
	<2–3 times/month	0.324	0.082–1.272	0.262	0.063–1.085

Bold values indicate statistical significance (*p* < .05).

### 3.4 Dietary intake and anxiety symptoms

This study also estimated the effects of dietary intake on anxiety symptoms (Table 4). Regarding anxiety symptoms, we observed greater intake of green leafy vegetables, eggs, and egg products as protective factors. More specifically, after adjusting for potential confounders, we observed consuming salted and smoked food <2–3 times per month was protective against anxiety symptoms (OR: 0.181, 95% CI: 0.040–0.828) than consuming the same every day. Participants consuming eggs and egg products once a week were more likely to have anxiety symptoms (OR: 2.253, 95% CI: 1.049–4.837). Similarly, the likelihood of anxiety symptoms in the participants consuming green leafy vegetables <2–3 times per month was 3.988 times higher (95% CI: 1.327–11.985) compared with women consuming the same once or more per day. The fully adjusted binary logistic regression model had goodness‐of‐fit using the Hosmer–Lemeshow test (χ^2^ = 7.445, *p* = .489).

**TABLE 4:** The association between anxiety symptoms and dietary intake.

Variables	Groups	Model 1		Model 2	
		OR	95% CI	OR	95% CI
Cereals (rice and maize)	≥1 time/day	1		1	
	2–3 times /week	0.694	0.293–1.644	0.720	0.296–1.750
	1 times /week	**0.411**	**0.181–0.933**	0.439	0.191–1.009
Milk and milk products	≥1 time/day	1		1	
	2–3 times /week	**2.256**	**1.011–5.034**	2.193	0.966–4.979
	1 times /week	2.416	0.856–6.822	2.235	0.765–6.531
	<2–3 times/month	2.198	0.864–5.589	2.091	0.789–5.542
Eggs and egg products	≥1 time/day	1		1	
	2–3 times /week	2.071	0.991–4.328	**2.253**	**1.049–4.837**
	1 times /week	2.444	0.864–6.911	2.751	0.923–8.202
	<2–3 times/month	0.725	0.186–2.822	0.768	0.192–3.067
Fruits (fresh fruit and fruit juice)	≥1 time/day	1		1	
	2–3 times /week	0.578	0.225–1.480	0.469	0.177–1.241
	1 times /week	2.617	0.872–7.849	1.942	0.623–6.047
	<2–3 times/month	0.799	0.202–3.164	0.741	0.174–3.149
Green leafy vegetables	≥1 time/day	1		1	
	2–3 times /week	1.358	0.574–3.215	1.115	0.458–2.717
	1 times /week	0.797	0.199–3.191	0.703	0.168–2.939
	<2–3 times/month	**5.054**	**1.766–14.462**	**3.988**	**1.327–11.985**
Aquatic products (fish, shrimp, crab, shellfish)	≥1 time/day	1		1	
	2–3 times /week	1.605	0.514–5.013	1.867	0.582–5.989
	1 times /week	1.758	0.519–5.958	2.084	0.589–7.369
	<2–3 times/month	1.451	0.407–5.167	1.637	0.441–6.077
Bean and bean products	≥1 time/day	1		1	
	2–3 times /week	0.522	0.187–1.457	0.531	0.186–1.519
	1 times /week	0.668	0.216–2.068	0.585	0.181–1.893
	<2–3 times/month	0.589	0.188–1.848	0.534	0.163–1.746
Meat	≥1 time/day	1		1	
	2–3 times /week	0.824	0.371–1.832	0.978	0.425–2.251
	1 times /week	0.878	0.346–2.231	0.959	0.372–2.474
	<2–3 times/month	0.548	0.212–1.414	0.583	0.218–1.561
Salted and smoked food	≥1 time/day	1		1	
	2–3 times /week	0.544	0.115–2.564	0.359	0.070–1.835
	1 times /week	0.443	0.089–2.201	0.333	0.064–1.740
	<2–3 times/month	0.258	0.060–1.109	**0.181**	**0.040–0.828**

Bold values indicate statistical significance (*p* < .05).

## 4 DISCUSSION

Our study investigated the potential relationship between dietary intake and emotional problems among pregnant women through a community‐based survey. Our findings indicated that a higher intake of green leafy vegetables and eggs and eggs products, significantly reduced the likelihood of depression and anxiety symptoms. Additionally, lower intake of salted and smoked foods acted as protective factors against anxiety symptoms.

First, our study reported the prevalence of emotional symptoms among pregnant women. Overall, the prevalence of anxiety and depression symptoms was relatively lower than that reported by Ma et al. in Shanghai, China (Vitaloni et al., 2019), while, the incidence of depression was higher than that in another study involving 1220 pregnant women in Chongqing, China (Tang et al., 2019). There are several potential explanations for the low levels of anxiety and depression symptoms observed in our study. In fact, a significant portion of the participants in our study have a higher level of education. Previous studies have demonstrated that educational attainment is regarded as a protective factor against mental health symptoms (Vitaloni et al., 2019). Thus, higher level of education means, more knowledge, skills, and supportive resources to mitigate related emotional symptoms. Surely, the higher proportion of married and employed women in our study also indicated the relatively decent socioeconomic status (SES) of our study sample. This implies that good interpersonal support and personal resources bear positive consequences for psychological well‐being (Linder et al., 2020).

To our knowledge, this study was the first to report the significant impact of dietary intake on mental health symptoms during the third trimester of pregnancy in Chinese pregnant women. Indeed, scholars have also shown that nutrition affects brain function in pregnant women, especially neurotransmitters, which might be considered the predominant explanation for the relationship between emotional symptoms and dietary intake (Bodnar & Wisner, 2005). Our finding revealed that increased intake of eggs and egg products was a protective factor against anxiety and depression symptoms in pregnant women, which was consistent with a previous study by Baskin et al. (2017). Eggs and egg products contain abundant nutrients, such as choline and essential fatty acids, which are beneficial for pregnant women and the growth and development of the fetus (Lutter et al., 2018). Multiple studies have shown that egg consumption can control blood pressure and reduce risk for gestational diabetes mellitus (Hillier & Olander, 2017; Milajerdi et al., 2018). Additionally, our study showed that a higher intake of green leafy vegetables was associated with fewer emotional symptoms, which is supported by a previous survey (Holder, 2019). Studies have shown that consumption of magnesium‐rich foods, such as green leafy vegetables and beans, could help reduce anxiety and irritability (Aucoin et al., 2021). A study published in JAMA Psychiatry linked major depression to inflammation in the brain (Setiawan et al., 2015). Green leafy vegetables contain high amounts of vitamins A, C, E, and K as well as minerals and anti‐inflammatory phytochemicals (Roberts et al., 2016). Hence, it is suggested to further study the relationship between mental health and dietary intake of pregnant women, to provide different dietary patterns for pregnant women with different health status, so as to regulate the mental health status of pregnant women more effectively.

In our sample, less consumption of salted and smoked food was associated with a lower incidence of anxiety symptoms. Consumption of salted and smoked food is very common in a Chinese family. However, salted and smoked foods contain large amounts of salt and carcinogens, which are very harmful to pregnant women. Scholars have indicated that high salt intake causes adverse maternal and fetal outcomes involving high blood pressure in individuals, cardiac and renal structure injuries in newborns (Piecha et al., 2012; Seravalli et al., 2016). However, the evidence of association between salt consumption and mental health symptoms during pregnancy has been reported rarely. One study from Japan found that there was no association between the western dietary pattern (high salt consumption) and depression symptoms during pregnancy (Miyake et al., 2018). Another evidence showed that there was a positive relationship between depression and salt (Goldstein & Leshem, 2014). Similarly, little is known about the relationship between smoked food and mental health symptoms. One potential explanation for our results is that the proper functioning of the brain relies on a consistent and healthy supply of nutrients. These nutrients contribute to the effective operation of the central nervous system and can potentially influence mood through the synthesis of neurotransmitters (Zahedi et al., 2014). Yet, poor nutrient content in salted and smoked foods prevents the provision of those steady and healthy elements. Considering the widespread and harmful nature of pickled food, it is recommended that communities carry out early diet health education for pregnant women, pay attention to the eating habits of pregnant women with different characteristics, and give timely correction guidance and nutrition health education to maintain the physical and mental health of pregnant women.

Pregnant women are more susceptible to malnutrition because of significant changes in physiology, psychology, and lifestyle (Jiang et al., 2018). In other words, these changes would directly affect an individual's biomedical system and form the basis for depression and anxiety symptoms (Leung & Kaplan, 2009). Thus, pregnant women are paid more attention to the nutrition development of the fetus and intake various, due to food cravings during pregnancy, which might result in less adherence to healthy dietary choices (Duke et al., 2017). Namely, dietary intake patterns can help medical workers identify key pregnant women in need of mental health support. Healthy dietary intake intervention is a recommended approach to address mental health problems in pregnant women.

The strengths and limitations of this study must be acknowledged. First, a major strength of this study that it was based on a representative sample of community and inclusion of specific dietary intake of pregnant women. Therefore, the results could be generalized to populations with similar socioeconomic backgrounds. Further, the participants were interviewed by trained researchers to obtain more detailed and accurate dietary intake‐related information to reduce recall bias and reflect the actual dietary habits of pregnant women. Our study provided valuable evidence for community‐based primary care and guidance for healthy dietary intake during pregnancy. However, this study only assessed the unidirectional association between mental health symptoms and dietary intake, the bidirectional association between the two needs to be further explored. Additionally, the limited ability to infer causality based on a cross‐sectional study was one of the limitations. Lastly, with the diversification of food, takeaway foods were also commonly consumed by people which should be further investigated in future studies.

## 5 CONCLUSION

Healthy dietary intake, especially green leafy vegetables, eggs and egg products, may be more effective in reducing anxiety and depression symptoms in pregnant women. Salted and smoked food are not recommended for pregnant women because of the close association with anxiety symptoms. This study provides valuable evidence for dietary intake interventions in pregnant women to mitigate the risk of depression and anxiety symptoms.

## AUTHOR CONTRIBUTIONS

**Yuhan Yu:** Data curation (lead); formal analysis (lead); investigation (lead); methodology (lead); software (lead); writing – original draft (lead); writing – review and editing (lead). **Wensu Zhou:** Data curation (equal); investigation (equal); methodology (equal); software (equal); writing – original draft (equal); writing – review and editing (equal). **Xidi Zhu:** Data curation (equal); software (equal); writing – original draft (equal); writing – review and editing (equal). **Zhao Hu:** Conceptualization (equal); investigation (equal); methodology (equal); writing – original draft (equal). **Shaojie Li:** Conceptualization (equal); formal analysis (equal); investigation (equal); methodology (equal); writing – original draft (equal). **Baohua Zheng:** Data curation (equal); formal analysis (equal); investigation (equal); methodology (equal). **Huilan Xu:** Supervision (equal); validation (equal); visualization (equal). **Wei Long:** Resources (equal); software (equal); supervision (equal). **Xiyue Xiong:** Methodology (equal); software (equal); supervision (equal).

## FUNDING INFORMATION

None.

## CONFLICT OF INTEREST STATEMENT

None.

## ETHICS STATEMENT

The Ethics Committee of the Xiangya School of Public Health (ID: XYGW‐2019‐056) approved this study.

## Data Availability

The raw data supporting the conclusions of this article will be made available by the authors without undue reservation.
